# Biomarkers in aneurysmal subarachnoid hemorrhage: A short review

**DOI:** 10.1016/j.wnsx.2023.100205

**Published:** 2023-04-22

**Authors:** Sávio Batista, Jhon E. Bocanegra-Becerra, Bernardo Claassen, Felipe Rubião, Nicollas Nunes Rabelo, Eberval Gadelha Figueiredo, Dan Zimelewicz Oberman

**Affiliations:** a Faculty of Medicine, Federal University of Rio de Janeiro, Rio de Janeiro, Brazil; b School of Medicine, Universidad Peruana Cayetano Heredia, Lima, Peru; c Department of Neurosurgery, University of São Paulo, São Paulo, Brazil; d Department of Neurosurgery, Hospital de Força Aérea do Galeão, Rio de Janeiro, Brazil

**Keywords:** Aneurysmal subarachnoid hemorrhage, Subarachnoid hemorrhage, Biomarkers, Neuroinflammatory markers, Aneurysm

## Abstract

Poor outcomes of aneurysmal subarachnoid hemorrhage (aSAH) can be the result of the initial catastrophic event or the many acute or delayed neurological complications. Recent evidence suggests that some molecules play a critical role in both events, through some unknown pathways involved. Understanding the role of these molecules in these events could allow to improve diagnostic accuracy, guide management, and prevent long-term disability in aSAH. Here we present the studies on aSAH biomarkers present in current medical literature, highlighting their roles and main results.

## 1 Introduction

Intracranial aneurysms are abnormal vascular dilations that occur in 3%–5% of the general population, characterized by localized damage to the arterial wall, with loss of the internal elastic lamina and alteration of the middle layer.^1^ The most dangerous cerebral aneurysm consequence is its rupture, with a morbidity and death rate of more than 50%. Aneurysmal subarachnoid hemorrhage (aSAH) survivorship rates range from 15 to 35% of patients with some form of lifelong impairment, cognitive deficit, or personality change.^2^

Poor outcomes of aSAH can be the result of the initial catastrophic event or due to the many acute or delayed neurological complications, such as cerebral ischemia, hydrocephalus, and re-bleeding.^3^ Recent evidence suggests that some molecules play a critical role in both events, through some still unknown pathways involved.^4^ Understanding the role of these molecules in these events could allow us to improve diagnostic accuracy, guide management, and prevent long-term disability.

Here we propose a short review of current studies about different biomarkers for aSAH presented in human studies, translating from their physiological point of view to their potential clinical usefulness.

## 2 Methods

### 2.1 Literature search

A systematic review of the literature for biomarkers in aSAH was conducted in accordance with the PRISMA guidelines. The terms “Subarachnoid hemorrhage AND Markers”, “Subarachnoid hemorrhage AND Biomarkers” or “Subarachnoid hemorrhage AND Neuroinflammatory markers” were used for the search. The search terms were queried using Pubmed, Embase, Scopus, Cochrane, and Web of Science databases.

#### 2.1.1 Inclusion and exclusion criteria

Literature in all languages that presented Biomarkers to the present day were considered. We included only those studies which discussed the Biomarkers in aSAH in clinical studies with patients. Non-English papers, letters to the editor, and commentaries were also excluded from the initial review.

## 3 Results

**Fig. 1:**
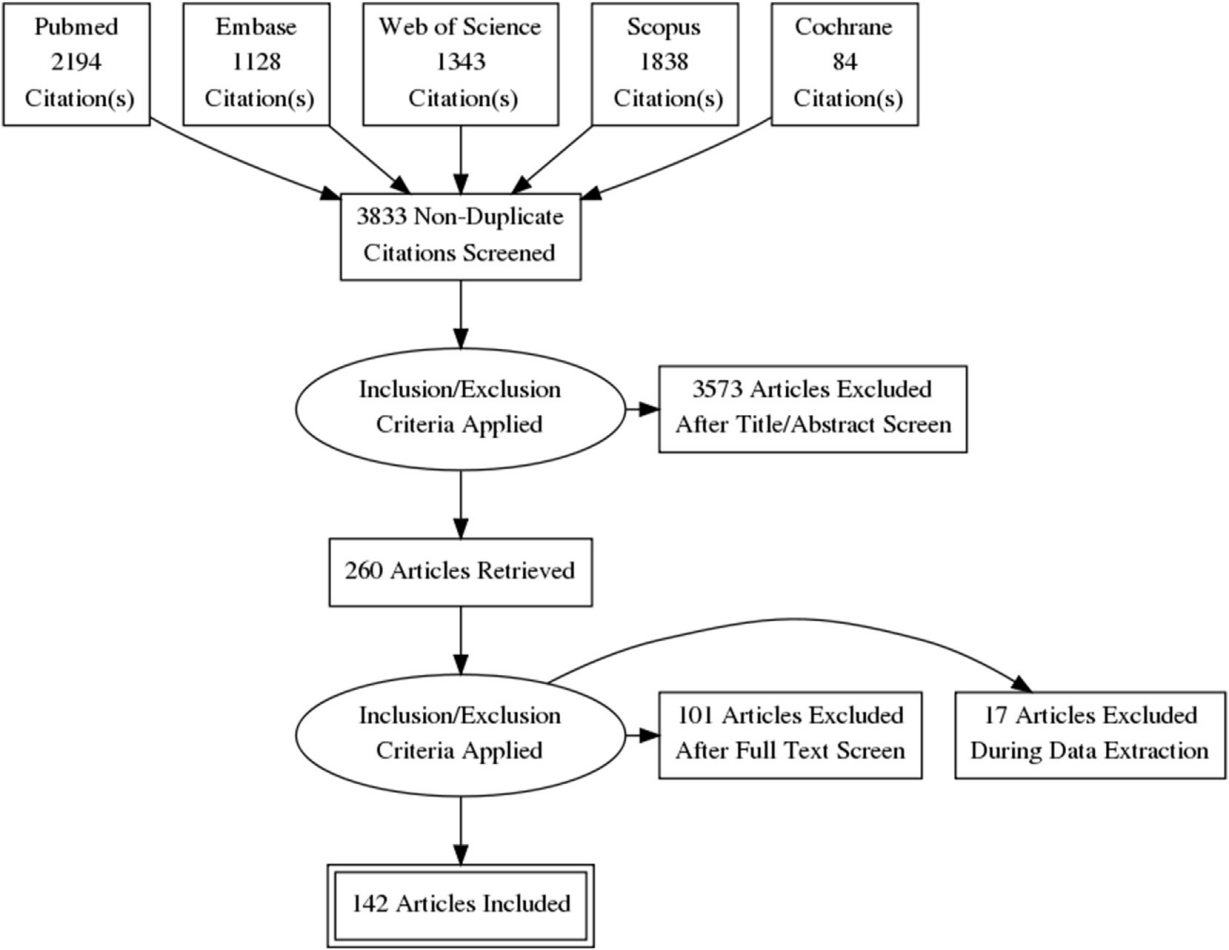
PRISMA Flow Diagram for biomarkers in aSAH.

We found 6587 articles, with 2194 in PubMed, 1128 in Embase, 1343 in Web Of Science, 1838 in Scopus, and 84 in Cochrane databases. Of these, 2754 were removed as duplicates. Titles of 3833 studies were screened manually, of which 51 were letter/commentaries, and 3100 other articles were excluded by title. A total of 682 were selected after Reading the abstract and 260 articles were available for a full-text review. Next, 101 articles were excluded as per our exclusion criteria, and 17 were during the data extraction. Finally, 142 studies were included in this review. The search is described in Fig. 1.

The final selection included 106 articles, including 103 observational prospective and retrospective studies, 1 randomized trial, and 2 reviews. The most commons biomarkers found and articles numbers they described were Blood components (29), Cytokines (17), MicroRNAs (11), C-reactive protein (CRP) (10), Lactate (7), Electrolytes (6), Cholesterol/lipid proteins (6), Glucose (5), and S100B (5). Table 1 provides a summary of the predictions for each biomarker associated with aSAH that have been reported in more than one study.

**Table 1:** Current predictive on aSAH biomarkers reported in the literature.

Biomarker	Predictive	Biomarker	Predictive
Lactate	Vasospasm, outcome, mortality, DCI	Hemoglobin	Vasospasm, outcome, DCI
C-reactive protein	Vasospasm, outcome, DCI	Heme oxygenase 1	Vasospasm, outcome
Glucose	Vasospasm, outcome, mortality, DCI	Osteopontin	Outcome
Copeptin	Vasospasm, outcome	HMGB1	Vasospasm, outcome, DCI
Neurofilament proteins	Outcome, mortality	F2-Isoprostanes and Isofurans	Vasospasm, outcome, DCI
S100B	Vasospasm, outcome, DCI	Kallikrein	Outcome, mortality
MicroRNAs	Vasospasm, outcome, DCI	UCH-L1	Outcome
Estrogen	Outcome, DCI	Periostin	Outcome, DCI
Alpha-II spectrin breakdown products	Vasospasm	Adhesion involved molecules	Vasospasm, outcome, DCI
Galectins	Outcome, DCI	GFAP	Outcome, DCI
Cholesterol/lipid proteins	Vasospasm, DCI	Aminoacids	Vasospasm, outcome, mortality
Caspase	Outcome, mortality	Cytokines	Vasospasm, hydrocephalus, DCI
D-dimer	Outcome, DCI	Electrolytes	Vasospasm, outcome, DCI
Tau	Outcome	Blood components	Vasospasm, outcome, mortality, DCI
Neuroglobin	Outcome, mortality, DCI	YKL-40	Outcome

DCI: Delayed Cerebral Ischemia; CSF: Cerebrospinal Fluid.

## 4 Discussion

### 4.1 Lactate

Lactate is produced in the central nervous system through aerobic glycolysis by astrocytes and is transferred to neurons to act as an alternative substrate for glucose during cerebral insult.^5^ Hyperlactatemia is common in the early phase of aSAH and is independently associated with hospital mortality and poor neurological outcome.^6^ Van Donkelaar et al described the maximum early serum lactate levels in the acute phase after aSAH associated with an increased risk for delayed cerebral ischemia (DCI)-related infarction and poor outcome, in the cutoff value at 1.6 mmol/L.^7^ Multivariable analysis controlling for Hunt and Hess grades showed lactic acid levels on admission to be an independent predictor of mortality with *p* = 0.0018.^8^

The aSAH may initiate immune interactions leading to decreased immune function, followed by an increased risk of infection. Radolf et al documented early elevated cerebral lactate correlated with the occurrence of bacterial pneumonia, while late elevations with delayed ischemic neurological deficits after aSAH.^9^ In the same way, elevated cerebrospinal fluid (CSF) lactate within the first 10 days following symptom onset correlates with the unfavorable outcome at discharge, not having any correlation between vasospasm and infection in a multivariate model, although there was a trend toward significance in a univariate analysis related to vasospasm.^10^ Lindgren et al found no associations at any time between CSF-lactate levels and impaired cerebral circulation or unfavorable outcomes, with CSF-lactate >2.1 mmol/L. Only >61 years of age and treatment with endovascular coiling were associated with a CSF-lactate above a reference level of >2.1 mmol/L in the acute phase after SAH (*p* < 0.04).^11^

Admission serum lactic acid was positively associated with Hunt-Hess grade and modified Fisher grade, glucose, troponin I, and white blood cell counts, and negatively associated with Glasgow Coma Scale (GCS) and ventilator-free days according to Poblete et al,^12^ but associated with the development of vasospasm or DCI. Patients with elevated lactic acid more often died during hospitalization, and less often were discharged home. However, after adjusting for other predictors of poor outcome, the adjusted odds of inpatient mortality (OR 0.97, 95% CI 0.79–1.20; *p* = 0.80) and discharge to home (OR 1.00, 95% CI 0.80–1.26; *p* = 0.97) was not associated with admission lactic acid.

#### 4.1.1 C-reactive protein

Inflammation is regarded to be an important factor influencing outcome after aSAH and has been demonstrated to play a role in early brain injury after aSAH, predicting poor outcome at 6 months independently of sepsis in a multivariate model, with baseline C-reactive protein (CRP) level of 17.5 mg/L on day 1 post ictus presenting 58% of sensitivity and 79% of specificity, whereas on day 3 post ictus, a threshold of 20.5 mg/L predicting poor outcome with a confidence level of 65% sensitivity and 68% specificity.^13^ Moreover, at admission, clinical vasospasm was significantly correlated with higher CRP levels (correlation coefficient methodology; *z* = 7.921, *p* < 0.0001, *r* = 0.75), and patients with hemodynamic changes in transcranial Doppler showed higher CRP levels than patients without vasospasm (correlation coefficient methodology; *z* = 8.421, *p* < 0.0001, *r* = 0.88). Patients with higher Hunt-Hess grades on admission developed significantly higher serum CRP levels (correlation coefficient methodology; *z* = 6.941, *p* < 0.0001, *r* = 0.81).^14^ Juvela et al relate CRP levels with the outcome, but these levels do not seem to predict DCI or infarction after aSAH.^15^ Whereas Jeon et al. Demonstrated that 1 or 2 days postoperative CRP levels were associated with the time profile of developing symptomatic vasospasm (*p* < 0.05).^16^

Clinical and radiologic severity of aSAH correlated significantly with serum CRP levels since patients with low GCS scores (*z* = −8.912, *p* < 0.0001, *r* = −0.89) and high Hunt-Hess (*z* = 6.467, *p* < 0.0001, *r* = 0.82) and Fisher grades (*z* = 7.652, *p* < 0.0001, *r* = 0.86) presented elevated serum CRP levels.^17^ Fountas et al showed similar results, and also angiographic vasospasm (*p* < 0.0001) correlation with serum and CSF elevated CRP levels. In addition, the CRP levels in serum and CSF were associated with poor clinical outcomes.^18^ Yang et al reported that at admission elevated baseline serum high-sensitivity-CRP level and antibiotic therapy were significant factors independently associated with acute kidney injury following aSAH (OR = 1.2, 95% confidence interval (CI) = 1.1–1.3, *p* = 0.003). Serum high-sensitivity-CRP level might be helpful as a predictor for the development of acute kidney injury, the sensitivity and specificity are 76.5% (13/17) and 64.6% (95/147), respectively, based on the threshold of 6.6 mg/L.^19^

## 5 Glucose

Many factors stimulate the sympathetic nervous system in aSAH patients, with catecholamine and cortisol release, both hormones related to blood glucose controls. The connection between high glucose levels and aSAH was shown in the literature. McGirt et al demonstrate that patients with persistently high blood glucose levels were seven-fold more likely to have a poor prognosis than patients with good glucose control, although an isolated hyperglycemic event was not a predictor of poor outcome.^20^

Glucose levels are also correlated with potassium, hemoglobin, and phosphate, although the pathophysiological mechanism remains unclear. Serum glucose/potassium ratio (GPR), was correlated with the incidence of cerebral infarction due to cerebral vasospasm and vasospasm grade.^21^ Jung et al found that GPR could predict mortality in aSAH patients at admission, (cutoff value of 37.8 for the GPR). Sensitivity and specificity for predicting 3-month mortality were 90.2 and 51.0%, respectively.^22^ Fujiki et al reported that serum GPR, glucose, and potassium level at admission were significantly correlated with poor outcomes at 3 months.^23^ The glucose-phosphate ratio at admission is associated with WFNS grade, a poor outcome at 3 months following aSAH, vasospasm (*r* = 0.581, *p* < 0.001), and DCI (*r* = 0.523, *p* < 0.001).^24^

Glycemic gap (GG), as determined by the difference between glucose and the hemoglobin A1c (HbA1c)-derived estimated average glucose (eAG). Sun et al found that admission GG was not independently associated with in-hospital mortality or poor outcome in a population of aSAH. The aGG cutoff value of ≥30 mg/dL had a sensitivity of 0.90 (95% CI 0.79, 1.0) and specificity of 0.41 (95% CI 0.31, 0.51) for mortality. For poor composite outcomes at the same cutoff value of 30 mg/dL, the sensitivity and specificity were 0.79 (95% CI 0.68, 0.90) and 0.45 (95% CI 0.33, 0.58), respectively.^25^

### 5.1 Copeptin

Copeptin is a peptide of 39 amino acids and is identified as the C-terminal component of pro-arginine vasopressin which is discharged along with pro-arginine vasopressin as the precursor peptide is processing, reflecting the pituitary-mediated stress response.

Copeptin has been proposed as a prognostic marker in many acute illnesses, such as aSAH. In aSAH, Copeptin levels increase with severity. Levels of copeptin have been reported to predict prognosis and symptomatic cerebral vasospasm in aSAH patients.^26^ This biomarker level on admission was statistically significantly associated with the severity of aSAH according to the WFNS grade, the amount of subarachnoid blood, and intracranial hemorrhage.^26^^,^^27^ The cutoff value of the plasma level of copeptin as an indicator for predicting poor outcome was projected to be 24.0 pmol/l, which yielded a sensitivity of 70.5% and a specificity of 69.6%, with an area under the curve (AUC) analysis of 0.74 (95% CI, 0.67–0.81), showing a significantly greater discriminatory ability to predict poor outcomes as compared with age.^28^

#### 5.1.1 Neurofilament proteins

Neurofilament proteins (NF) are components of the axonal cytoskeleton, expressed exclusively in neurons. NF subunits differ in molecular weight—NF-H (heavy), NF-M (medium), and NF-L (light), with the light unit of the neurofilament protein (NFL) being the primary component of the neurofilament core. It has been demonstrated that NFL in CSF can be used as a marker of neuronal damage, particularly axonal degeneration.^29^ NF is consistently elevated in the CSF and blood of aSAH patients, and some studies indicate that this elevation may be associated with disease severity on admission to the hospital, long-term outcomes, and increased mortality rate.^30^, ^31^, ^32^, ^33^

### 5.2 S100B

S100B is a calcium-binding protein not only expressed in astroglia, thus S100B elevations can also be related to muscle lesions or orthopedic trauma without head injury.

Abboud et al observed high S100B serum levels with higher Hunt-Hess grade and poor outcomes during the first 3 days after hemorrhage.^34^ Brain damage related to vasospasm occurs in most cases after day 7. Therefore, monitoring S100B blood levels over a longer period might lead to improved outcome prediction. Based on it, Weiss et al reported a mean 8-day blood level after aSAH as effective in predicting the outcome.^35^ Late ischemia due to vasospasm (after the first week) can occur. Sanchez-Peña et al found that levels at 15-day also may be used as predictors. The S100B cutoff was not modified by the presence of an intracerebral hematoma secondary to aSAH, and only the 15-day S100B value significantly predicted the outcome (*p* < 0.0005). The best cutoff value was 0.23 ug/L (specificity 0.90, 95% CI 0.81–0.95; sensitivity 0.91, 95% CI 0.75–0.98).^36^ The mean CSF S100B level in patients without cerebral vasospasm was much lower than that in patients with cerebral vasospasm. The CSF S100B level of different time points and the mean CSF S100B level can predict the risk of cerebral vasospasm using univariate analysis. Mean CSF S100B or S100B levels at different time points in patients without cerebral vasospasm were much lower than that in patients with this one, which suggested the potential role of S100B as an important prognostic factor of cerebral vasospasm.^37^

Despite these previous studies highlighting the relation between serum and CSF S100B to demonstrate current and prediction outcomes, Kiiski et al in his study found no association between serum S100B and outcome. However, serum S100B levels were higher in patients with non-severe initial clinical presentation (WFNS 1–3, *p* = 0.007), suggesting that S100B might have an adaptive role in brain injury after aSAH.^38^

### 5.3 MicroRNAs

MicroRNAs (miRNAs) are implicated in the cellular metabolism of inflammatory responses in the central nervous system.^39^ Especially important evidence has been accumulated regarding the potential role in predicting clinical outcomes in patients with ruptured brain aneurysms.

Wang et al identified seven miRNAs in CSF as reliable predictors of delayed cerebral vasospasm, which included Let-7b-5p, miR-15b-5p, miR-17–5p, miR-19b-3p, miR-20a-5p, miR-24–3p, and miR-29a-3p. This panel achieved a risk prediction of 87% for cerebral vasospasm in a testing cohort (AUC = 1).^40^ In an experiment conducted by Lu et al, a panel of 4 miRNAs (miR-4532, miR-4463, miR-1290, and miR-4793) allowed to differentiate aSAH patients with DCI from those without DCI with an AUC of 100% (95% CI 1.000–1.000, *p* < 0.001). In addition, the capability to distinguish aSAH patients with or without DCI from healthy controls was observed, with an AUC of 99.3% (95% CI 0.977–1.000, *p* < 0.001) and 82.0% (95% CI 0.685–0.955, *p* < 0.001), respectively.^41^

Pulcrano-Nicolas et al reported that “hsa-miR-3177–3p” was significantly associated with the risk of vasospasm, with higher levels in patients who developed vasospasm vs. those who did not (6.20 ± 0.47 vs. 5.62 ± 0.61).^42^ Stylli et al reported a difference in the expression of miRNAs in CSF samples (miR-27a-3p, miR-516a-5p, miR-566, and miR-1197) between the vasospasm and non-vasospasm aSAH groups.^43^ Su et al compared miRNAs from peripheral blood cells of patients with SAH to healthy control, miRNA-132 and miRNA-324 showed an upregulation in both aSAH DCI and non-DCI groups. However, differences between the aSAH DCI and non-DCI groups were not statistically significant. Besides, it was shown that miR-132 presented a 9.5 fold (95% CI: 2.3 to 16.7) upregulation in the aSAH DCI group and 3.4 fold (95% CI: 1.0 to 5.8) upregulation in the non-DCI group. From the ROC curve of miR-324, the AUC was 0.97 for the DCI group and 0.96 for the non-DCI group. miR-132 gave an AUC of 0.75 for the DCI group and 0.73 for the non-DCI group.^44^

Sheng et al reported the usefulness of miR-1297 as a prognostic indicator of aSAH. Serum levels from blood collected at 24 and 72 h enabled the prediction of the neurological outcome at 1 year with AUC of 0.8 (95% CI: 0.73–0.86; *p* < 0.001) and 0.94 (95% CI: 0.90–0.97; *p* < 0.001), respectively.^45^ In another study, Sheng et al investigated the temporal changes in miR- 502–5p expression after aSAH and the time to peak level. Multivariate logistic regression revealed that higher miR-502–5p levels at 7 days were associated with a significantly high risk for poor outcome, based on a Modified Rankin Scale (mRS) 3–5 value, post-aSAH. On day 7, the AUC was 0.97 (95%CI, 0.931–0.990, *p* < 0.001) with a sensitivity of 95.3% and specificity of 92.5%.^46^

### 5.4 Estrogen

Ramesh et al found that postmenopausal women with low-estradiol levels and with the TT genotype of PvuII variant in the ESR1 gene are at 3.5- folds higher risk of aSAH (CI 95% 1.424–8.828, *p* = 0.0074).^47^ Crago et al provided the first clinical evidence that plasma E1 and E2 concentrations are associated with the severity of injury and outcomes after aSAH. Higher E1 and E2 levels were associated with higher Hunt-Hess grade (E1, *p* ¼ 0.01; E2, *p* ¼ 0.03), the presence of DCI (E1, *p* ¼ 0.02; E2, *p* ¼ 0.02), and poor 3-month outcomes (E1, *p* ¼ 0.002; E2, *p* ¼ 0.002). Trajectory analysis identified distinct populations over time for E1 (61% E1 high) and E2 (68% E2 high). Patients in higher trajectory groups had higher Fisher grades (E1, *p* ¼ 0.008; E2, *p* ¼ 0.01), more frequent DCI (E1, *p* ¼ 0.04; E2, *p* ¼ 0.08), and worse 3-month outcomes (E1, *p* ¼ 0.01; E2, *p* ¼ 0.004) than low groups.^48^

### 5.5 Alpha-II spectrin breakdown products

The breakdown products of alpha-II spectrin (SBDPs), a cytoskeletal protein, could be regarded as important biomarkers in aSAH. Lewis et al reported that both calpain- and caspase-mediated a-II spectrin breakdown product levels are significantly increased in patients suffering from aSAH. The concentration of SBDPs was found to increase significantly over baseline level up to 12 h before the onset of cerebral arterial vasospasm.^49^

Papa et al reported significant differences in SBDP150, SBDP145, and SBDP120 CSF levels between patients with and without aSAH (*p* < 0.001). The AUC for distinguishing aSAH from control subjects was 1.0 for SBDP150 and SBDP145, and 0.95 for SBDP120. Besides, SBDP150 and SBDP145 both yielded sensitivities and specificities of 100%, and SBDP120 was 90% and 100%, respectively.^50^

### 5.6 Galectins

Galectins are b-galactoside-binding lectins involved in several cellular processes, including brain inflammatory responses.^51^ In particular, evidence suggests that galectin-3 might represent an important predictor marker in the history of aSAH. Nishikawa et al reported that a galectin-3 cutoff value of 3.48 ng/ml predicted DCI development or poor outcome (specificity 70.6%; sensitivity 73.3%). Consequently, proposing its usefulness to predict the development of DCI.^52^ Also, the associations between galectin-3 and the severity and poor prognosis following aSAH were studied by Liu et al, who suggested that Gal-3 may aid in predicting the risk of poor prognosis in patients with aSAH, with a sensitivity of 81.1%, specificity of 77.1% and AUC = 0.84 (95%CI, 0.762–0.9).^53^

### 5.7 Cholesterol/lipid proteins

Lipoprotein-associated phospholipase A2 (Lp-PLA2) is an endothelial inflammatory marker with a defined role in the pathogenesis of atherosclerosis, however, it might also represent a key factor in aSAH.^54^ For instance, Lp-PLA2 predicted the risk of vasospasm with a sensitivity and specificity of 65.4% and 86.3%, respectively, and AUC = 0.798 (95% CI = 0.712–0.883), based on a threshold of 187.6 μg/L.^55^ Moreover, Lp-PLA2 obtained within the first 24 h post-aSAH predicted symptomatic cerebral vasospasm in patients with aSAH.^56^ Similarly, the serum level of Lp-PLA2 was significantly elevated in patients with DCI and decreased within the first 2 days after admission. Thus, Lp-PLA2 in the early stages of aSAH might be a novel predictive biomarker for the occurrence of DCI.^57^

On the other hand, oxidized low-density lipoprotein (ox-LDL) and its receptor, lectin-like ox-LDL receptor-1 (LOX-1) have been proposed as potential aSAH biomarkers, this is because the union of LOX-1 and Ox-LDL results in the reduction of nitric oxide availability and initiates the inhibition of vasodilation.^58^^,^^59^ Lin et al reported that serum LOX-1 had a sensitivity of 76.2% and specificity of 80.7% to predict DCI in patients with aSAH (AUC 0.825, 95% CI 0.747–0.887).^60^

### 5.8 Caspase

The value of Caspase-3 collected on day 3 after aSAH predicted 6-month mortality and poor outcome.^61^ Inflammasome Caspase-1 allowed to differentiate between good and poor clinical outcomes after aSAH.^62^ Caspase-cleaved cytokeratin-18 predicted 6-month mortality and poor outcome after aSAH.^3^

### 5.9 D-dimer

D-dimer had a sensitivity and specificity of 82.09% and 78.43%, respectively in predicting poor outcomes at 6 months.^58^ D-dimer levels above 41.1 ng/ml in the first 72 h after aSAH had a sensitivity of 69.2% and specificity of 75% for predicting DCI. Thus, elevated D-dimer in these scenarios may be a potential predictive biomarker for DCI.^63^

### 5.10 Tau

Tau protein is a microtubule protein that builds the structural basis of the axonal cytoskeleton. It exists in six isoforms that enable the axoplasmic flow of proteins in the neurons. As a protein-restricted to brain intracellular compartments, acute axonal injuries may result in the liberation of the extracellular space. Some studies suggest axonal injury after sSAH and increased tau protein levels in the CSF,^31^ which could be used as a biomarker to predict neuropsychological outcomes.^64^

Helbok et al collected serial cerebral microdialysis (CMD) samples from 22 aSAH patients with multimodal neuromonitoring to determine CMD-total-tau by ELISA in the CSF. They reported an increased CMD-total-tau level ≥1259 pg/ml on day 2 discriminated between bad (mRS ≥4) and good (mRS 0–3) functional outcome (sensitivity of 80% and a specificity of 77%, AUC-ROC curve, mean = 0.76 (0.55–0.98), *p* = 0.04).^20^ These results suggest that tau protein levels at CSF may be an important biomarker for predicting bad outcomes in patients with aSAH.

### 5.11 Neuroglobin

Neuroglobin is a molecule that increases oxygen availability to brain tissue and provides protection under hypoxic or ischemic conditions. The predictive performance of day 3 neuroglobin level for the occurrence of DCI was represented as AUC = 0.77 (95% CI = 0.69–0.86), and the sensitivity, specificity, and Youden index were derived as 73.9%, 72.5%, and 0.46, respectively, based on the best threshold of 8.4 ng/ml.^65^

Serum neuroglobin on day 2 significantly predicted 6-month mortality with 9.03 ng/ml, with the highest sensitivity of 86.7%, highest specificity of 81.4%, and highest AUC of 0.893 (95% CI = 0.812–0.974, *p* < 0.05). Also, it predicted a 6-month unfavorable outcome with values > 9.03 ng/ml), the highest sensitivity of 76.5%, and the highest specificity of 80.5% (AUC = 0.818, 95% CI = 0.691–0.954, *p* < 0.05).^66^

### 5.12 Hemoglobin

Anemia can be a predictor of some variables in aSAH patients. The optimal cutoff value for hemoglobin (Hb) level as a predictor for acute epilepsy after aSAH was determined as 119 g/L in the ROC curve (sensitivity was 75%, and specificity was 69.48%).^67^ Oxyhemoglobin (OxyHb) levels in CSF showed a delayed peak reaching the highest levels in the high-risk period for developing delayed cerebral ischemic between days 3 and 14 after aneurysm rupture. Patients with delayed ischemic neurological deficits had a significantly higher cumulative oxyHb exposure within the first week after bleeding.^68^ A Hb concentration of 9 g/dL is associated with an increased incidence of brain hypoxia and cell energy dysfunction in patients with poor-grade aSAH.^69^ Lower Hb levels are associated with worse outcomes regardless of aSAH severity or the development of vasospasm.^70^

### 5.13 Heme oxygenase 1

Heme-oxygenase 1 (HO-1) is a microsomal enzyme released by cell stressor triggers and may play a role as an anti-inflammatory molecule. Patients who developed sonographic vasospasm and DCI had significantly higher HO-1 expression levels than those who developed vasospasm, but no DCI.^71^ Wang et al identified a clinically useful HO-1 cutoff value of 81.2 μM in identifying patients with an unfavorable outcome (64.7% sensitivity, 100% specificity, 100% positive predictive value, 80.0% negative predictive value, and 82.4% accuracy for HO-1 > 81.2 μM as a predictor of unfavorable outcome).^72^

### 5.14 Osteopontin

Osteopontin (OPN) levels are associated with central nervous system pathologies. Their levels on days 10–12 were the most useful predictor of poor outcome at cutoff values of 915.9 pmol/L (sensitivity, 0.694; specificity, 0.845, AUC = 0.815).^73^ OPN levels, in CSF and plasma, displayed a weak correlation on day 1 and were higher, in CSF, at all time points. Only in poor prognosis patients, OPN levels in CSF significantly increase on day 4 and day 8. Plasma OPN on days 1 and 4 was a predictor of poor outcomes.^74^

### 5.15 HMGB1

High-mobility group box 1 protein (HMGB1) is a nuclear protein that can be actively secreted by microglia or passively released by necrotic neurons, serving as a possible biomarker in aSAH damage, with DCI being associated with his levels along hospitalization,^32^ and level at admission may be a predictive biomarker for cerebral vasospasm.^75^

### 5.16 F2-Isoprostanes and isofurans

F2-Isoprostanes (F2-IsoPs) and isofurans (IsoFs) are isomers of prostaglandins formed by free radical-induced peroxidation of arachidonic acid. High levels of urine F2-IsoPs have already been associated with DCI, cerebral vasospasm, and further clinical conditions.^76^^,^^77^ IsoFs may represent a specific biomarker predicting DCI following aSAH.^78^

### 5.17 Kallikrein

Serum Kallikrein (KLK) emerges as a potential biomarker for assessing hemorrhagic severity and predicting DCI following aSAH.^79^ A reference interval for KLK6 was established by using serum samples (*n* = 136) from an adult population. Additionally, serum samples (*n* = 326) from patients with aSAH (*n* = 13) were collected for 5–14 days, to study the concentration of KLK6 in this disease. The correlation between KLK6 and S100B, an existing brain damage biomarker, was analyzed in 8 of 13 patients. The reference interval for KLK6 was established to be 1.04–3.93 ng/ml. The mean levels in patients with aSAH within the first 56 h ranged from 0.27 to 1.44 ng/ml, with the lowest levels found in patients with worse outcomes. There were significant differences between patients with good recovery or moderate disability (*n* = 8) and patients with severe disability or death (*n* = 5) (mean values of 1.03 ng/ml versus 0.47 ng/ml, respectively) (*p* < 0.01). There was no significant correlation between KLK6 and S100B. Decreased serum concentrations of KLK6 are found in patients with aSAH, with the lowest levels in patients who died.^80^

### 5.18 UCH-L1

Ubiquitin C-Terminal Hydrolase L1 (UCH-L1) is a member of the deubiquitinating enzymes named Ubiquitin C-Terminal Hydrolases, usually present exclusively on neurons and neuroendocrine cells, concentrated on the perikarya and dendrites. Its presence in the CSF usually reflects central nervous system injuries and the destruction of those structures. In aSAH patients, UCH-L1 levels had no meaningful difference between patients with good and poor outcomes on the first-day post–AR, according to the mRS scale. On day 5, it was observed that baseline levels of UCH-L1 were more elevated in patients with poor outcomes (mRS 3–6) than in patients with good neurological outcomes (mRS 0–2) (*p* = 0.001),^81^ with the same pattern being detectable using the Glasgow Outcome Scale Extended (GOS-E). Although its overexpression is a predictive factor for a more negative outcome (GOS-E 0–1), levels of UCH-L1 had no significant difference in patients with GOS-E 3–8.^82^

### 5.19 Periostin

Periostin (POSTN) is a 90 kDa extracellular matrix protein that is closely related to inflammation and believed to play a role in the pathological process after aSAH. Luo et al reported that serum POSTN had an association with the patient's outcome, with higher levels in patients with a poor outcome when compared with ones with a good outcome at 12 months (328.48 ± 121.40 vs 186.30 ± 85.69, *p* < 0.01).^83^ Also, POSTN levels were higher in patients who developed DCI in the first 6 days, peaking between days 4 and 6, but the difference was lost when CSF drainage was performed. POSTN levels on days 1–3 showed to be a predictor of DCI when above the 80.5 ng/ml value (95% CI, 1.900–299.000; *p* = 0.014). These patients had higher levels of CRP when compared to those who didn't go through CSF drainage, with no correlation to the levels of POSTN.^84^

### 5.20 Adhesion involved molecules

Adhesion molecules are molecules that mediate the interaction between cells, or between cells and extracellular matrix. Selectins are molecules associated with circulating cells to endothelium. Von Willebrand factor (VWF) is a large protein with an important role in the platelet adhesion process that is secreted primarily by vascular endothelial cells but can also be produced by platelets in reduced amounts. One of VWF's main regulators is ADAMTS13 (a disintegrin-like and metalloprotease with thrombospondin type 1 repeats), an enzyme that is responsible for reducing its activity by cleaving VWF. VWF and Selectins are adhesion molecules associated with adhesion.

Tang et al have shown that aSAH patients who developed DCI not only had considerably higher levels of VWF but also of IL-6 and P-selectin, both molecules involved primarily in cell adhesion (*p* < 0.05). The concentrations of these molecules were measured on days 1, 4 ± 1, and 9 ± 1. Also, the activity of ADAMTS13 was reduced on day 1 in patients who developed DCI in comparison with the control group (*p* < 0.05). VWF had lower concentrations in patients with a good outcome when compared to those who had a poor outcome in all 3 timespans (*p* < 0.05).^85^ Serum VWF was also related to cerebral vasospasm along with Vascular Endothelial Growth Factor and Matrix Metalloproteinase-9. These molecules had increased concentrations in patients before cerebral vasospasm when compared to those who did not develop this condition (*p* < 0.023). Elevated levels of at least two of these markers showed a positive predictive value of 100%.^86^

### 5.21 GFAP

GFAP is an intermediate filament protein that isn't present in the peripheral blood circulation in normal circumstances, being released in situations of glial injury with the breakdown of astrocytes' skeletons and consequent damage to the blood–brain barrier. A study from Tatli et al has shown that GFAP on the CSF was higher in patients who developed aSAH when compared to the control group (*p* < 0.001). The CSF samples were taken between 2 and 13 h from the onset of symptoms in the aSAH group, without a significant correlation between the onset of symptoms and sample collection (*r* = −0.06, *p* = 0.69). The difference wasn't observed when analyzing serum GFAP levels. The ROC curve AUC for CSF GFAP for aSAH diagnosis was 0.761 (95% CI = 0.652–0.850; *p* < 0.001), and the best cutoff value is 8.5 ng/ml with a sensitivity of 58.5% (95% CI = 42.1–73.7), and specificity 92.1% (95% CI = 78.6–98.2).^87^

On the other hand, serum GFAP fraction levels were increased in the subsequent days in those patients with any sort of secondary events, such as ischemia or re-bleeding, related to aSAH (i.e. last value/first value) (*p* = 0.0012), also being related to major focal deficits and less favorable outcomes (*p* < 0.001).^88^

### 5.22 Aminoacids

Sokol et al showed that on days 0–3 after aSAH onset, patients with a poor outcome had CSF levels of nine amino acids significantly higher than those with good outcomes: taurine (*p* = 0.038), aspartic acid (*p* = 0.038), citrulline (*p* = 0.035), glutamic acid (*p* = 0.038), gamma-amino-butyric acid (*p* = 0.043), 3-methyl-histidine (*p* = 0.01), ornithine (*p* = 0.033), cystathionine (*p* = 0.01), and isoleucine (*p* = 0.045), but it was observed that, on day 5, there only were increased levels of glutamic acid (*p* = 0.041).^89^

Also, there was a significant difference in Arginase-1, an enzyme that indicates hemolysis and produces Nitric Oxide, in the CSF of patients that developed cerebral vasospasm in both pre (*p* = 0.029) and during the vasospasm onset phases (*p* = 0.038). Its activity was also measured by the l-arginine/l-ornithine ratio, which was significantly lower in patients who developed cerebral vasospasm when compared to the control group (*p* < 0.0001) and patients with aSAH that did not develop this one (*p* = 0.0009).^90^ It was also observed that patients with lower Reactive Hyperemia Index had bigger chances of dying in the first 30 days after aSAH (*p* = 0.07), but it was not related to any sort of neurological deficit.^91^

### 5.23 Cytokines

IL-6 is a cytokine with hormone-like activity with an important role in vascular and metabolic diseases. Serum IL-6 levels had a positive correlation with delayed ischemic neurological deficits on all days after aSAH complications, except day 1, and IL-6 was also positively correlated with cerebral vasospasm at days 3 (*p* = 0.025), 7 (*p* = 0.012), 9 (*p* = 0.014) and 13 (*p* = 0.031) and hydrocephalus at days 9 (OR = 8.1, *p* = 0.025, 95% CI = 1.3–50.1) and 13 (OR = 9.6, *p* = 0.025, 95% CI = 1.3–69.3).^92^ CSF IL-6 was also related significantly with DCI (mean day 4–14 peak, DCI: 26,291 ± 24,159 pg/ml vs. no DCI: 16,184 ± 13,163 pg/ml; *p* = 0.023).^93^ It had good potential for diagnosis of ventriculitis when compared to patients who developed aSAH alone, with a cutoff value of 3100 ​pg/ml (AUC ​= ​1.00, cutoff ​= ​707 ​pg/ml, sensitivity ​= ​100%, specificity ​= ​100%, +LR ​= ​∞; -LR ​= ​0), and to indicate it when compared to cerebral vasospasm patients and SAH alone patients (sensitivity ​= ​86.7%, specificity ​= ​82.1%, +LR ​= ​4.8, -LR ​= ​0.16). Also, it had the moderate diagnostic potential for differentiating ventriculitis from aSAH vasospasm (AUC ​= ​0.757, cutoff ​= ​3100 ​pg/ml, sensitivity ​= ​86.7%, specificity ​= ​70.6%, +LR ​= ​2.9, -LR ​= ​0.19).^94^^,^^95^ A peak value of IL-6 above 10,000 pg/ml was also correlated with a higher incidence of shunt-related hydrocephalus (*p* = 0.009), with other risk factors such as aneurysm location on the anterior cerebral artery and its branches or in the posterior circulation (*p* = 0.025), and age ≥60 years (*p* = 0.014).^96^

In parallel to IL-6, aSAH patients also had increased levels of other inflammatory cytokines, such as IL-1β (*p* < 0.05), IL-18 (*p* < 0.05), and TNF-α (*p* < 0.01) when compared to non-SAH patients, and higher levels were observed in patients with lower WFNS scores (*p* < 0.0001) and higher Hunt-Hess grade (III-IV, *p* < 0.0001) and Fisher grades 3–4 (*p* < 0.001). Also, higher levels of these cytokines were related to brain edema, hydrocephalus, and cerebral vasospasm (*p* < 0.01; *p* < 0.01 and *p* < 0.01, respectively).^97^

### 5.24 Electrolytes

aSAH patients presented important variations in magnesium levels, an element that is important for cell and body function maintenance. A state of hypomagnesemia, i.e. 2.8 (2.7 and 2.9) mg x dl(-1) was observed in patients who underwent DCI when compared to non-DCI cases, which had concentrations of 2.9 (2.8 and 3.0) mg x dl(-1) (*p* < 0.05).^98^ Patients who underwent MgSO4 therapy showed a different S100B curve when compared to the control group, which might indicate that it has some neuroprotective potential. The MgSO4 had a median value of 225 pg/ml followed by a slow decrease, while the control group showed a peak with a median of 392 pg/ml (*p* = 0,04), meaning that this group had higher biomarkers of glial damage.^30^

Hyponatremia is also a common disturb in aSAH patients, and those who suffer from it have a reported 65.1% chance of developing cerebral vasospasm, while normonatremia patients have a 28.5% chance of doing so (OR 6.25, 95% CI [2.96–13.19]; *p* < 0.0001). Hyponatremia also has a positive correlation to a poor clinical outcome (GOS<4) in comparison to normonatremia patients (OR 2.78, 95% CI [1.33–5.83]; *p* = 0.0057) and longer hospital stay (*p* < 0.0001). However, it had no correlation between hyponatremia and the development of DCI.^99^^,^^100^

Hypokalemia has a common imbalance, with some studies suggesting high catecholamine level causes overactivation of the Na+/K ​+ ​-ATPase pump, which causes a shift in potassium ions from extracellular to intracellular spaces. Tam et al found that neither hypokalemia nor hyperkalemia was an independent predictor for poor outcomes 3 months after SAH onset.^101^ By contrast, other studies found that only 2% of the patients included were hypokalemic on emergency department admission, and hypokalemia in the subacute phase (days 7–10) correlated with poor outcomes at 3 months after discharge.^102^ Thus, the effect of a low potassium level on the outcome in aSAH patients remains controversial.

### 5.25 Blood components

Some blood component results seem to correlate with aSAH, being a possible element of accuracy, shown by sensibility/specificity. DCI was predicted by the Neutrophil/Lymphocyte ratio (87.3%/48.4%, and 63%/53%), and Platelet/Lymphocyte ratio (55.3%/78.5%).^103^ Vasospasm risk was predicted by Platelets (40%/90.5%, and 78%/85%), Lymphocytes (57.9%/83.3%), Neutrophils (40%/100%), Leukocytes (50%/100%), and Combined Platelet and Leukocytes high values (60%/89.5%).^104^^,^^105^ Rebleeding had an association with the Neutrophil/Lymphocyte ratio (72.3%/63.3%), and the Neutrophil/Lymphocyte ratio combined with Fisher Grade (39.94%/100%).^106^ Poor outcome correlated with Neutrophil/Lymphocyte ratio (74.5%/69.3%), and Platelet/Lymphocyte ratio (55.3%/78.5%).^107^ Mortality in 3 months appears predicted by RDW value (80.6%/91.6%).^108^

### 5.26 YKL-40

Elevated levels of YKL-40, a glycoprotein involved in the activation of the innate immune system and in cell processes in relation to extracellular matrix remodeling, were found in patients with aSAH in both cerebrospinal fluid and serum than controls.^109^ However, Kacira et al. Demonstrated that YKL-40 levels are not correlated with the severity of subarachnoid hemorrhage and cannot be used as a serological marker of inflammation in patients with an aneurysm rupture.^110^

### 5.27 Other biomarkers

Other aSAH biomarkers with only one study reported were summarized in Table 2.

**Table 2:** Summary of Other Biomarkers for aSAH.

Biomarker	Predictive	Biomarker	Predictive
NOX4^111^	Outcome, DCI	Leptin^128^	Outcome, mortality
Gelsolin^112^	Outcome, mortality	Netrin-1^129^	Outcome
Microalbuminuria^113^	Outcome	Neuropeptide Y^130^	Vasospasm
Visfatin^114^	Mortality	Adrenomedullin^131^	Outcome, mortality
Tumor necrosis factor-like weak inducer of apoptosis^115^	Outcome	Thioredoxin^132^	Outcome, mortality
pituitary adenylate cyclase-activating polypeptide^116^	Outcome, mortality	sCD40L^133^	Outcome, mortality
RhoA^117^	Vasospasm	Myeloperoxidase^134^	Vasospasm
Metalloproteisase-9^118^	DCI	Calprotectin^135^	Outcome, DCI
Vasopressin and Oxytocin^119^	Outcome	Biomarkers of Glycocalyx Injury^136^	DCI
Thrombospondin-1^120^	Vasospasm	sST2^137^	Mortality, DCI
cDPP3^121^	Outcome, DCI	Cardiac Troponin I^138^	Outcome
Acrolein^122^	DCI	MBP^139^	Outcome
EET genetic markers^123^	Outcome, DCI	Mitochondrial membrane potential^140^	DCI
TNFSF14 and OSM^124^	DCI	SERPINE1 gene^141^	Outcome, DCI
Metabolomic profile^125^	DCI	Metallomics of CSF^142^	Outcome, mortality
Haptoglobin^126^	DCI	Nitric Oxide^143^	Vasospasm, outcome
Fibrinogen^127^	Outcome	Taurine^144^	DCI

DCI: Delayed Cerebral Ischemia; CSF: Cerebrospinal Fluid.

## 6 Conclusions

The use of biomarkers as a predictor of severity, complications, and prognosis could improve the management of aSAH patients. We listed and discussed the main biomarkers in the aSAH scenario reported in the current medical literature, and although there are some gaps related to the accuracy, some biomarkers are demonstrating promising results and could be helping patients in the near future.

## Funding

No financial support.

## Declaration of competing interest

The authors have no personal, financial, or institutional interest in any of the drugs, materials, or devices described in this article.

## Abbreviations list

aSAH: Aneurysmal Subarachnoid Hemorrhage
DCI: Delayed Cerebral Ischemia
CSF: Cerebrospinal Fluid
GCS: Glasgow Coma Scale
CRP: C-reactive Protein
GPR: Serum glucose/potassium ratio
GG: Glycemic gap
eAG: estimated Average Glucose
AUC: Area Under the Curve
NF: Neurofilament proteins
NFL: Light unit of neurofilament protein
miRNAs: MicroRNAs
mRS: Modified Rankin Scale
SBDPs: Alpha-II spectrin breakdown products
Lp-PLA2: Lipoprotein-associated phospholipase A2
ox-LDL: oxidized Low-density Lipoprotein
LOX-1: Lectin-like ox-LDL receptor-1
CMD: Cerebral Microdialysis
Hb: Hemoglobin
OxyHb: Oxyhemoglobin
HO-1: Heme-oxygenase 1
OPN: Osteopontin
HMGB1: High-mobility group box 1 protein
F2-Isoprostanes: F2-IsoPs
IsoFs: Isofurans
KLK: Serum Kallikrein
UCH-L1: Ubiquitin C-Terminal Hydrolase L1
GOS-E: Glasgow Outcome Scale Extended
POSTN: Periostin
VWF: Von Willebrand factor
